# The Effect of Electrical Stimulation of the Calf Muscle on Leg Fluid Accumulation over a Long Period of Sitting

**DOI:** 10.1038/s41598-017-06349-y

**Published:** 2017-07-20

**Authors:** Daniel Vena, Jonathan Rubianto, Milos R. Popovic, Geoff R. Fernie, Azadeh Yadollahi

**Affiliations:** 1 Toronto Rehabilitation Institute, University Health Network, Toronto, Canada; 2 Institute of Biomaterials and Biomedical Engineering, University of Toronto, Toronto, Canada

## Abstract

Leg fluid accumulation during sedentary behaviours such as sitting can lead to leg edema and associated adverse health consequences. This study investigates the use calf muscle electrical stimulation (ES) to reduce seated leg fluid accumulation. Thirteen non-obese, normotensive men (mean age 51 yr.) with sleep apnea were enrolled in the study. Participants first lay supine for 30 minutes to equalize fluid distribution and then sat for 150 minutes. While seated, participants received either active or sham ES of the calf muscles, according to random assignment. Participants returned one-week later to cross over to the other study condition. Leg fluid was measured continuously while sitting using the bioelectrical impedance method. Fluid accumulation in the leg was reduced by more than 40% using active ES, compared to sham ES (∆ = 51.9 ± 8.8 ml vs. ∆ = 91.5 ± 8.9 ml, P < 0.001). In summary, calf muscle ES is an effective method for reducing accumulation of fluid during long sedentary periods and has potential use as a device for preventing leg edema to treat associated health consequences in at-risk groups and settings.

## Introduction

Fluid accumulation in the legs is a known consequence of sedentariness and is associated with edema formation. Development of leg edema is associated with complications including increased risk of leg ulcers, painful swelling, reduced elasticity of the veins and arteries of the legs, and thromboembolic events^1, 2^. More fluid in the legs is also associated with a greater shift of fluid toward the upper-body when moving from upright to supine posture^3^, which can increase risks for supine hypertension^4^, acute decompensation in heart failure^5^, pulmonary edema^6^, nocturnal asthma^7^, and increased sleep apnea severity^8^. This is particularly problematic in settings that facilitate sedentariness such as the office^9^ or long-haul flights^10^, and in high risk populations such as the spinal cord injured^11^ and individuals with fluid-retaining diseases^1^. Reducing leg fluid accumulation can serve to prevent the genesis or worsening of these diseases and disorders in high risk populations and settings^11–15^.

During upright sitting or standing, fluid accumulates in the legs by first rapidly filling the capacitance vessels of the lower body^16–18^. According to the Starling equation, fluid filters from vasculature into the tissue spaces when capillary pressure is increased and/or tissue pressure is decreased^19^. Fluid continues to shift from the upper to the lower body by displacing fluid that filters into the interstitial spaces of the leg, driven by the enhanced capillary transmural pressure^20–22^. Conversely, fluid is reabsorbed into the vasculature when tissue pressure is increased and/or capillary pressure is reduced.

Compression stockings are a commonly prescribed and effective therapy for reducing leg fluid by increasing tissue pressure. However, they are limited by their discomfort and difficulty to apply and remove, contributing to their low adherence^23^. In addition, improper fit is a common problem, which increases the risk of developing exudative skin lesions^24^ and deep vein thrombosis^25^. Alternatively, activation of the skeletal pump in the legs reduces leg fluid by reducing capillary pressure in the legs and increasing tissue pressure^26^. Walking is one method of activating the skeletal pump, however, it is limited to able-bodied individuals and is less feasible for the elderly or those with mobility limitations. Therefore, in the present study, we experiment with activation of the skeletal pump using electrical stimulation (ES) of the gastrocnemius muscles to prevent leg fluid accumulation while seated.

The overall purpose of this study was to investigate the efficacy of calf muscle ES in preventing leg fluid accumulation while seated for two-and-half hours. The main objectives of this study were first, to determine if calf muscle ES can prevent the accumulation of leg fluid over the seated period; and second, to identify the minimum time period required for active calf muscle ES to reduce seated leg fluid volume. A secondary objective, which was exploratory in nature, was to characterize the temporal changes in leg fluid accumulation with and without calf muscle ES.

## Results

As part of a larger study examining the effect of calf muscle ES on fluid shift and sleep apnea, one hundred and thirty-two participants were pre-screened for eligibility and 45 participants moved on to be screened for sleep apnea. From this group, 15 participants were found to have moderate to severe sleep apnea (apnea-hypopnea index = 21.1 ± 4.0 events/hr) and were willing to participate in the full study. One participant was found to have varicose veins and was therefore excluded, and another was lost to follow-up, yielding a final sample size of 13. Weight, height, and blood pressure were measured at a screening visit to confirm participant eligibility. Although sex was not an exclusion criterion, all participants were non-obese men with a mean age of 51 ± 2 years and body mass index (BMI) of 26.5 ± 0.7 kg/m^2^. Participants were all normotensive with a mean resting systolic blood pressure, diastolic blood pressure, mean arterial pressure, and heart rate of 117.5 ± 3.2 mm Hg, 80.4 ± 2.6 mm Hg, 93.0 ± 2.5 mm Hg, and 68.3 ± 2.9 beats/min. All participants were free from symptoms of any serious diseases or disorders.

The study protocol required participants to first lay supine for 30 minutes to equalize fluid distribution and then sit for 150 minutes on two separate days spaced one week apart. On one of the two study days, seated participants received either active or sham ES. Active ES elicited repeated calf muscle contractions in both legs every two seconds, whereas in sham ES the participant could feel the electrical current but it did not elicit calf muscle contractions. The protocols for the sham and active ES conditions were identical in every aspect (e.g. device and electrode type and position), except that in the sham ES condition electrical current was set to a level where the participant could feel the electrical current but a contraction was not elicited. After the first study day, participants returned one-week later to cross over to the other study condition. Leg fluid from ankle to knee was measured in the dominant leg continuously while sitting using the bioelectrical impedance method. Baseline leg fluid volume at the start of sitting did not differ between the sham (1306.3 ± 63.9 ml) and active (1370.8 ± 56.9 ml) ES conditions (P > 0.10). Similarly, baseline calf circumferences measured at the start of sitting were also similar between the sham (38.9 ± 0.8 cm) and active (39.1 ± 0.8 cm) ES conditions (P > 0.10).

Leg fluid volume increased significantly over the seated period in both the sham ES condition (∆ = 91.5 ± 8.9 ml, P < 0.001) and in the active ES condition (∆ = 51.9 ± 8.8 ml, P < 0.001). However, the interaction effect demonstrated that leg fluid volume increased significantly more in the sham ES condition compared to the active ES condition (P < 0.001). Calf circumference was measured in the dominant leg at the start and end of the seated period. Calf circumference increased significantly over the seated period in the sham ES condition (∆ = 0.79 ± 0.1 cm, P < 0.05), but was unchanged in the active ES condition (∆ = 0.10 ± 0.1 cm, P > 0.10). The interaction effect was significant, demonstrating the increase in calf circumference in the sham ES condition was significantly greater than the change during the active ES condition (P < 0.001).

**Figure 1:**
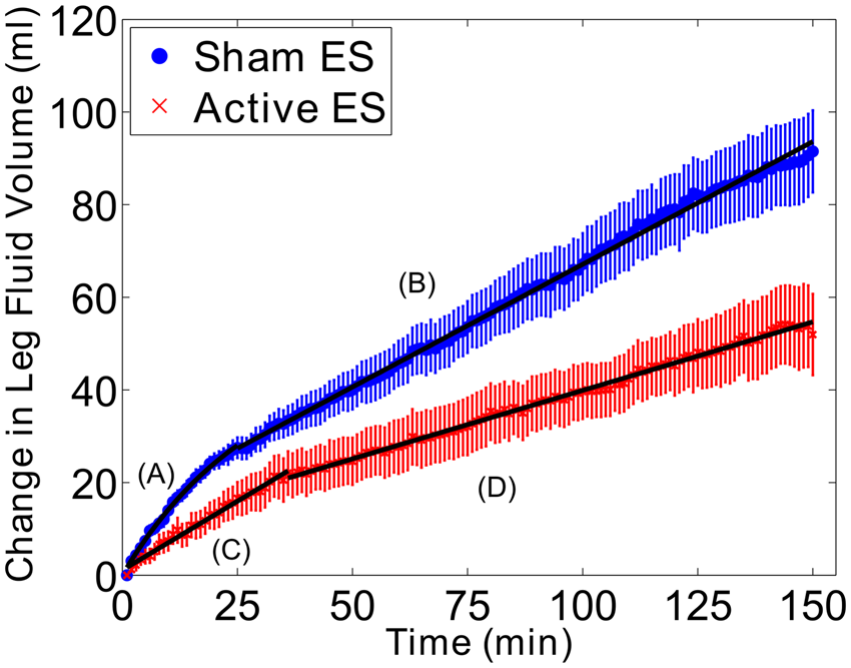
Mean change in leg fluid volume over the 150 minutes seated period in the active (red) and sham (blue) ES study conditions with standard error bars. Temporal pattern of fluid accumulation was different between the active and sham ES study conditions, with fluid accumulating more rapidly in sham ES study condition. The temporal patterns of fluid accumulation were also different between the initial and latter portions of sitting. The models representing the initial and latter portions of siting in both the active and sham ES conditions are described by equations A through D. A: *LFV*(*t*) = 34.0 (1 − *e* ^−0.03*t*^). B: *LFV*(*t*) = 0.53*t* + 26.8. C: *LFV*(*t*) = 0.60*t* + 1.1. D: *LFV*(*t*) = 0.30*t* + 20.7.

**Figure 2:**
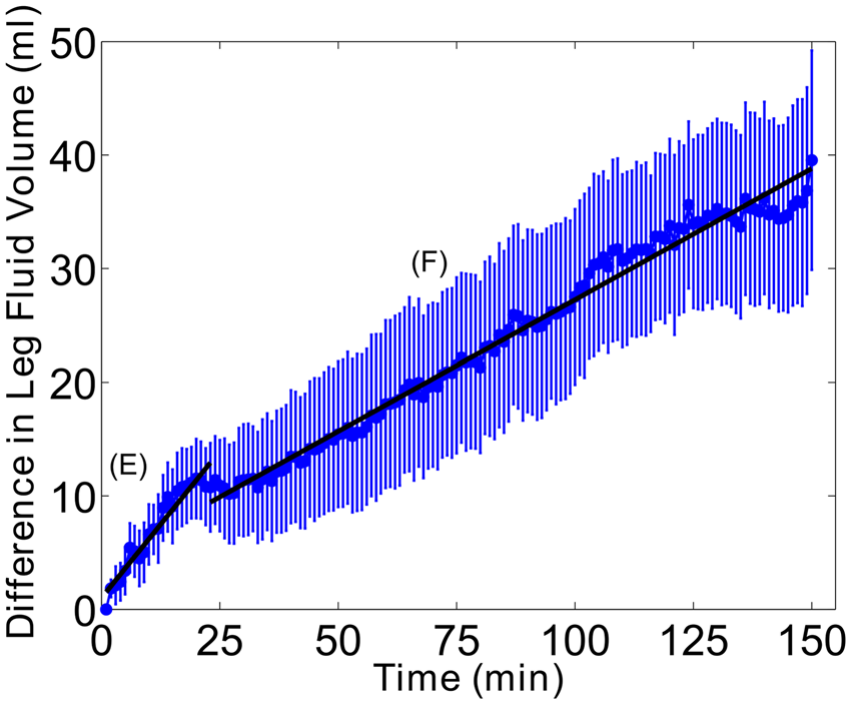
Mean difference in leg fluid accumulation between the active and sham ES conditions over the 150 minute seated period with standard error bars. The temporal pattern of differences in leg fluid accumulation between study conditions shows steady increase as sitting time progresses, and the rate of increase was different between the initial and latter portions of sitting. The models representing the initial and latter portions of the difference in fluid volume are described by equations E and F. F: *LFV*(*t*) = 0.52*t* + 1.0. E: *LFV*(*t*) = 0.23*t* + 9.2.

The temporal patterns of leg fluid accumulation in the active and sham ES conditions are displayed in Fig. 1. The temporal change in the difference in leg fluid accumulation between active and sham ES conditions is illustrated in Fig. 2. All temporal fluid data consisted of two distinct portions. Each portion was modeled as either a linear or exponential decay, based on model fit (see equations 2 and 3 in the Methods section). Model equations and the corresponding portions of temporal data they represent are described in Figs 1 and 2. In the sham ES condition, leg fluid initially accumulated according to an exponential decay function (Fig. 1, equation A), with a point of inflection at 25 minutes. After 25 minutes, leg fluid increased linearly, with a slope of 0.53 ml/min (Fig. 1, equation B). In the active ES condition, leg fluid increased according to two linear functions, with a point of inflection at 33 minutes. Up to the 33 minute time point, fluid increased linearly with a slope of 0.60 ml/min (Fig. 1, equation C). After 33 minutes, fluid also increased linearly but with a smaller slope of 0.30 ml/min (Fig. 1, equation D).

As illustrated in Fig. 2, the temporal pattern of difference in leg fluid accumulation between active and sham ES conditions continues to increase as sitting time progresses. The mean difference in leg fluid accumulation at the end of the 150 minute period between active and sham ES conditions was 40.0 ± 9.7 ml. The rate of increase of differences between sham and active ES conditions occurs linearly throughout the seated period in two distinct portions separated at the 23 minute time point and differentiated by their slopes (Fig. 2, equations E and F). As illustrated, the slope of the line in the initial portion (0.52 ml/min) is greater than the slope of the line in the latter period (0.23 ml/min).

**Figure 3:**
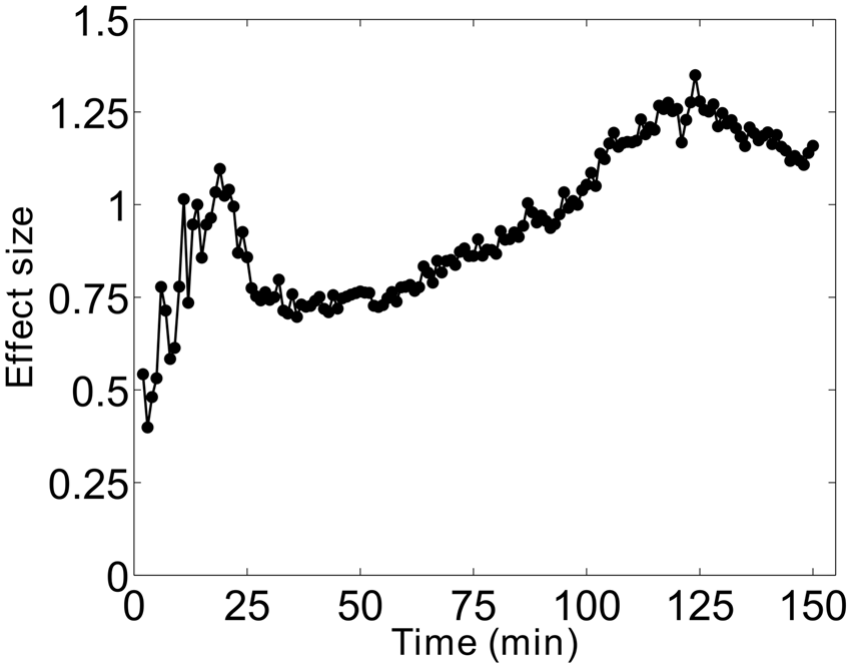
Cohen’s *d* effect size of volume difference over the 150 minutes seated period. Effect size is computed as the mean difference divided by the standard deviation of differences. Overall, effect size rises throughout the entire period seated, with a distinct peak at 20 minutes driven by the sharp rise in the difference in leg fluid accumulation and low variability.

The difference in leg fluid accumulation between active and sham ES conditions also demonstrated a rise in the variability among subjects (Fig. 2) at the 20 minute time point. This was best illustrated with the effect size, shown in Fig. 3, which takes into account the magnitude of the difference as well as the variability. As shown, there was a distinct peak in the effect size at 20 minutes when mean difference in leg fluid accumulation was relatively high and the variability was low. This was followed by a sharp drop in the effect size due to the rise in variability. The effect size gradually increased again as differences between active and sham ES continued to increase, while variability remained relatively stable (Fig. 3).

**Figure 4:**
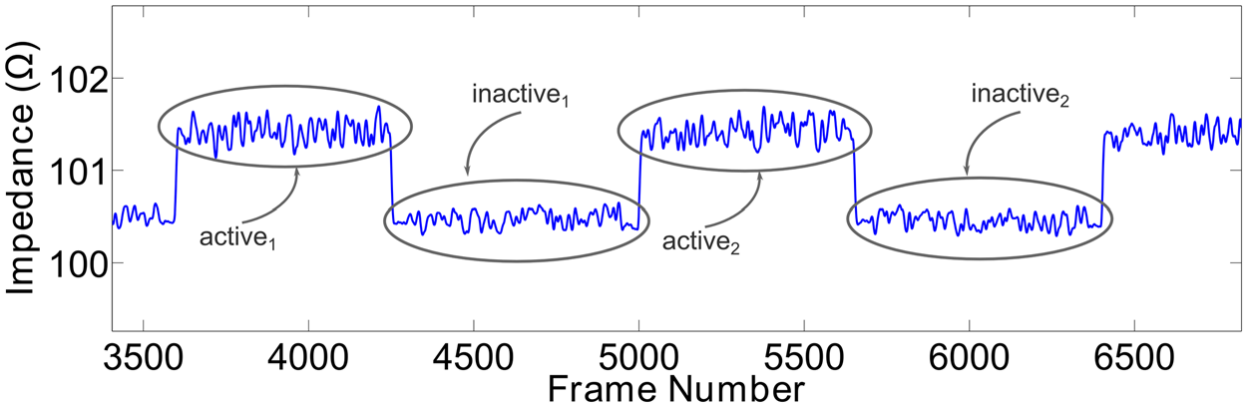
Representative data showing the active and inactive portions of two contractions. As illustrated, impedance increases with contraction (active) representing fluid ejection from the legs, and then drops when there is no contraction (inactive).

**Figure 5:**
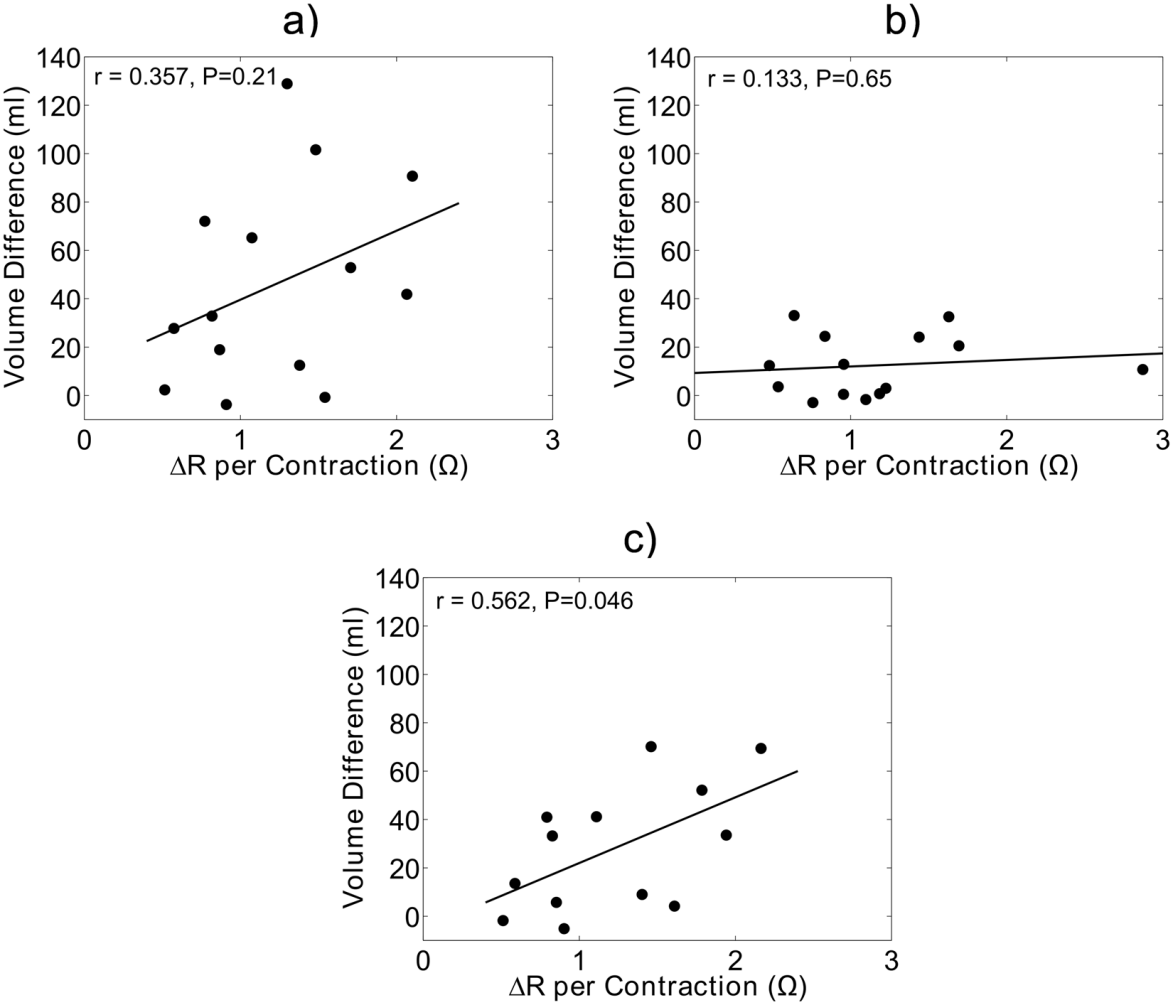
Scatterplots illustrating correlations between final volume differences and the change in resistance per contraction (ΔR per contraction) for: (**a**) all study data (**b**) the first 20 minutes seated and (**c**) the latter 130 minutes seated. Correlations are assessed by either Pearson’s correlation coefficient or Spearman’s rho depending on normality of the data distribution as assessed by the Shapiro-Wilk test of normality.

To measure the efficacy of the electrical stimulator in removing fluid upon calf muscle contraction, the change in resistance (ΔR) of the leg per contraction was computed. Resistance of tissues is inversely related to fluid volume. Therefore, the sharp rises in resistance per contraction depicted in Fig. 4 represent the ejection of fluid from the leg per contraction. The correlation between the ΔR per contraction during active ES and final difference in leg fluid volume between the active and sham ES conditions is illustrated in Fig. 5. As shown in Fig. 5-a, there was no relationship between ΔR per contraction and the difference in leg fluid accumulation across the entire seated period. After data were separated into two data sets based on the transition point at 20 minutes, the data set from the initial 20 minutes showed no relationship between ΔR per contraction and final volume difference (Fig. 5b). However, in the latter 130 minutes there was a significant positive correlation between volume difference and change in resistance per contraction (Fig. 5c, P = 0.046).

## Discussion

The present study reports several key findings: first, electrical stimulation of the calf muscle reduced the accumulation of fluid in the legs by 43% over a two-and-a-half hour (150 min) seated period. Second, during the sham ES condition, leg fluid accumulated according to an exponential decay during the first 25 minutes of sitting, then linearly in the latter period of sitting. Third, variability in the difference in leg fluid accumulation between active and sham ES conditions increased substantially after 20 minutes of sitting. Lastly, over the final 130 minutes of sitting, ΔR per ES contraction was associated with the difference in leg fluid volume between the active and sham ES conditions.

The present study reports that calf muscle ES reduced leg fluid accumulation by 40 ml and reduced calf circumference by 0.7 cm. The clinical relevance for these results can be derived from prior studies investigating interventions to reduce leg fluid prior to sleeping and effectively reduce sleep apnea severity (as measured by the apnea-hypopnea index) by 27% to 36%^12, 13, 27^. A comparison of fluid volumes measured by bioelectrical impedance with prior studies is not possible because prior studies have predominantly measured fluid volume in the whole leg (from hip to ankle), while the present study measured fluid volume in the lower leg (from knee to ankle). In terms of calf circumference, wearing compression stockings for one week reduced evening (pre-sleep) calf circumference by 0.5 cm^13^, one day of ultrafiltration reduced evening calf circumference by 1.0 cm^27^, and four weeks of walking exercise caused no change in evening calf circumference^12^. Calf muscle ES performed comparably, reducing calf swelling by 0.7 cm, despite a short duration of use. This warrants further research to examine whether the long-term use (such as 4 weeks) of calf muscle ES can be used to treat sleep apnea by reducing leg fluid accumulation and the consequent fluid shift from the legs to the neck that occurs during sleep.

Few studies have investigated the immediate effect of calf muscle contraction on leg fluid while upright^26, 28^. To our knowledge the findings of the present study are the first to demonstrate the immediate capability of calf muscle contraction via electrical stimulation to reduce leg fluid accumulation while seated, compared to a sham control. The experiment by Goddard *et al*. compared 30 minutes of quiet sitting followed by 30 minutes of calf muscle activation using a plantar reflex–based stimulation device^28^. These results differed from those reported in present experiment, as Goddard *et al*. report no accumulation of leg fluid volume over the 30-minute quiet sitting period, and a sustained reduction in leg fluid over the 30-minute period with leg muscle contractions. Differences in the pattern of leg fluid accumulation can likely be attributed to the methodological differences between the studies. Specifically, leg fluid was measured using air plethysmography, only women were studied, and participants sat for 15 minutes prior to beginning leg fluid measurements which means that the majority of the vascular fluid accumulation was not recorded. Furthermore, since each seated period started with 30 minutes of quiet sitting, calf muscle pumping was tested after 45 minutes of quiet sitting. Therefore leg fluid volume was higher when muscle contractions began. As a result, their study investigates the removal of fluid already accumulated in leg, rather than prevention of leg fluid accumulation which was the purpose of present study.

Stick *et al*. measured changes in leg fluid volume using a mercury strain gauge around the largest circumference of the calf while participants were (i) recumbent for 30 minutes, (ii) upright for 10 minutes, (iii) quiet sitting for 15 minutes, then (iv) sitting while using a cycle ergometer for 20 minutes^26^. They found that calf circumference increased substantially while upright and seated. During cycling, the study demonstrated a strong effect, reducing calf circumference by approximately the same proportion that it increased during quiet sitting (i.e. complete reversal of leg fluid accumulation). In the present study, measures of calf circumference were also suggestive that active ES had a strong effect in resisting accumulation of fluid in the legs. Active ES of the calf muscle resulted in no significant change in calf circumference over the entire seated period, while sham ES resulted in a 0.79 cm increase in calf circumference. Caution must be used when comparing the efficacy of cycling and calf muscle ES, given the methodological differences between the studies. In particular, Stick *et al*. evaluated the reduction of leg fluid accumulation during calf muscle exercise after 10 minutes of standing and 15 minutes of quiet sitting. Therefore as previously explained, Stick *et al*. report the removal of fluid already accumulated, rather than prevention of leg fluid accumulation that was addressed in the current study.

During the first 25 minutes of quiet sitting, fluid accumulated according to an exponential decay function and at a more rapid rate than in the latter portion of quiet sitting (Fig. 1). Past studies have reported that the initial rapid accumulation of fluid was suggestive of rapid filling of the capacitance vessels in the lower body^16, 17^. However, rapid vascular filling while upright was complete after approximately 5 minutes^18, 21, 29^; much less than our reported 25 minutes. One explanation for the discrepancy in vascular filling time is differences in body position. In the present study, participants were sitting in a chair, while in the past studies participants were standing at varying degrees head-up tilt. Regardless of the discrepancy in the time point where rapid vascular filling was complete, it is clear that leg fluid accumulated according to two different functions separated at the 25 minute time point. Following the rapid vascular filling, further increase in leg fluid was considered to be driven by enhanced transmural pressure leading to fluid filtration into the interstitial space^20–22^. Therefore, the latter portion of sitting was likely a reflection of the accumulation of interstitial fluid.

The difference in fluid accumulation between the active and sham ES condition describes the reduction in leg fluid accumulation due to active ES while seated. As illustrated in Fig. 2, the difference in leg fluid accumulation between the sham and active ES conditions increased with time. However, the difference in leg fluid between the study conditions is not an accurate measure of the effectiveness of active ES because it does not take into account the variability across the participants. A better measure for this is the effect size, which is the mean difference normalized to the variability across the participants. A local peak in the effect size occurred at the 20 minute time point, illustrating that effectiveness of active ES at reducing leg fluid accumulation steadily increased up to the 20 minute time point (Fig. 3). The sharp drop at 20 minutes is the result of the increased variability and is indicative of active ES being less effective at reducing leg fluid in some of the participants. After the sharp drop, variability remained high. However, the effect size continued to rise in proportion to the continued increase in the mean difference in leg fluid accumulation. Altogether, the results suggest that there was a strong effect of ES in the first 20 minutes, but the longer the participant uses ES, the more effective it will be at reducing leg fluid while seated.

One plausible explanation for the rise in variability after the first 20 minutes is the type of fluid being removed. In the first 20 minutes, active ES was likely preventing vascular filling while in the latter 130 minutes, active ES had variable effectiveness in preventing fluid filtration into the interstitium. From this, we hypothesized that differences in contraction strength across the subjects might explain the variability in the effectiveness of active ES in preventing interstitial fluid accumulation. As illustrated in Fig. 5, higher volumes of leg fluid ejection per contraction (ΔR per contraction) were associated with greater differences in fluid accumulation between the active and sham ES conditions in the latter 130 minutes of sitting. These results demonstrate that consistently strong contractions causing high fluid ejection from the legs is required during the period using calf muscle ES to prevent accumulation of interstitial leg fluid.

The greatest feature of an electrical stimulator for activating the skeletal pump and reducing leg fluid accumulation is its versatility as a therapeutic device. An electrical stimulation system can be developed into a compact, wearable device that can be applied in a wide variety of settings, thereby making it easier to target individuals who are at risk for leg fluid accumulation and its negative consequences. At risk groups include sedentary office workers who experience leg swelling due to long periods sitting^9^; flight attendants or frequent flight travellers susceptible to leg swelling due to a long immobilized period^10^; individuals with fluid retaining diseases such as renal failure and heart failure^1^; spinal cord injured^11^; individuals with sleep apnea caused by nocturnal shift of fluid from the legs to the neck^12–15^; and individuals with asthma who experience worsening of asthma symptoms due to nocturnal fluid shift^7^.

The main limitation of this study was that vascular and interstitial fluid were not directly measured and inferences made on the transition from vascular to interstitial fluid accumulation are based on the rates of change in leg fluid described by their mathematical models. To confirm these findings, more objective measures should be considered. Indicator dilution methods are an objective measure of fluid compartments, however it is invasive, requiring blood samples for each time measurement^30^. Ultrasound is a non-invasive method of estimating blood volume in major veins and arteries that could be performed in future studies to detect time-series changes in blood volume^31^. Our study was also limited in its lack of women participants. While the study was open to women, fewer women responded to the study advertisement and those that did were not eligible for various reasons. The main challenge with enrolling women to participate was the requirement for a sleep apnea diagnosis, which is a more common disorder in men^32^. To the best of our knowledge, there are no studies investigating differences between the sexes in leg fluid accumulation while upright. However, we have shown in prior studies that fluid shift due to gravity when lying down differed between the sexes. In short, baseline LFV was greater in men compared to women, yet there was no significant difference between the sexes in fluid shift out of the legs when lying down for 90 minutes^3, 33^. In addition, while the volume of fluid leaving the legs was the same, less fluid accumulates in the thorax and neck of women compared to men, possibly due to fluid sequestration in the abdomen of women^33^. Since the same amount of fluid shifted out of the lower body in men and women, it is likely that when assuming the upright position the same amount of fluid will shift into the lower body. With respect to between-sex differences in the effectiveness of the calf muscle activation in reducing leg fluid accumulation, to the best of our knowledge this has never been investigated. So while it is unlikely that leg fluid accumulates differently in men compared to women, the effects of calf muscle ES between the sexes cannot be speculated upon, but would be an interesting topic for future study.

In conclusion, calf muscle ES was effective at reducing the accumulation of fluid in the legs while seated, compared to a sham control. There was no clear time point after which calf muscle ES stopped being effective. While effect size reached a local peak at 20 minutes and then dropped substantially, after continued use the effect size increased substantially to an absolute peak at 130 minutes. Therefore, the longer an individual used calf muscle ES, the more effective it was at preventing leg fluid accumulation. Efficacy of calf muscle ES was consistent in preventing vascular filling in the first 20 minutes seated, but variable in preventing interstitial fluid formation in the latter 130 minutes seated. The variability in preventing interstitial fluid formation was found to be attributed to the amount of fluid ejected from the leg per contraction, where more fluid leaving the leg per contraction was associated with greater prevention of interstitial leg fluid formation. This highlights the importance of maintaining a strong contraction throughout the sedentary period using calf muscle ES. Once validated in a larger sample size, calf muscle ES has potential as a device for preventing leg fluid accumulation and associated health consequences in at-risk groups and settings.

## Methods

### Participants

The protocol was approved by the Research Ethics Board of Toronto Rehabilitation Institute and participants provided written informed consent prior to participation. The study was performed in accordance with the approved guidelines and regulations. Criteria for study enrolment were adult men and women with a body mass index < 30 kg/m^2^, and blood pressure < 140/90 mmHg. The study was part of a larger study investigating the efficacy of calf muscle electrical stimulation on fluid shift and sleep apnea severity, so all participants had a positive screening for sleep apnea with an apnea-hypopnea index ≥ 10, as determined by a portable sleep monitoring device^34, 35^. Participants abstained from alcohol and coffee consumption for 24-hours. Participants were excluded if they reported a history of cardiovascular, renal, neurological, or respiratory disorders; reported taking prescribed medication for these disorders; or reported taking over-the-counter medication that might influence fluid retention. Participants were recruited from the community through advertisement.

### Measurements

Fluid volume was measured in the dominant leg continuously while seated using a bioelectrical impedance measurement system. This method is a non-invasive technique used to estimate fluid volumes of the tissues. The method has been well validated and is highly reproducible with an accuracy within 0.5% compared to reference measures of total body water, repeatability within 0.3%, and test-retest correlation >95%^36, 37^. The method is based on Ohm’s law which states that the resistance of tissues to electrical current is directly proportional to its length: R = ρL^2^/v, where ρ is the resistivity of the fluid, L is the segments length, v is the fluid volume and R is the measured resistance^38–40^. This formula has since been adapted to measure impedance of tissues in individual body segments (leg, abdomen, thorax, and neck) and calculated according to equation 1^33, 41, 42^.

1 $${\rm{v}}=\frac{{{\rm{\rho }}}^{2/3}}{3{(4{\rm{\pi }})}^{1/3}}{(\frac{{\rm{L}}}{{{\rm{C}}}_{1}{{\rm{C}}}_{2}{\rm{R}}})}^{2/3}{{\rm{L}}(C}_{1}^{2}+{{\rm{C}}}_{2}^{2}+{{\rm{C}}}_{1}{{\rm{C}}}_{2})$$

Where C1 and C2 are the circumferences of the top (knee) and bottom (ankle) of the segment, L is the length of the segment, R is the segments resistance, and ρ is the resistivity of the blood which is estimated as 98 Ωcm^43^.

**Figure 6:**
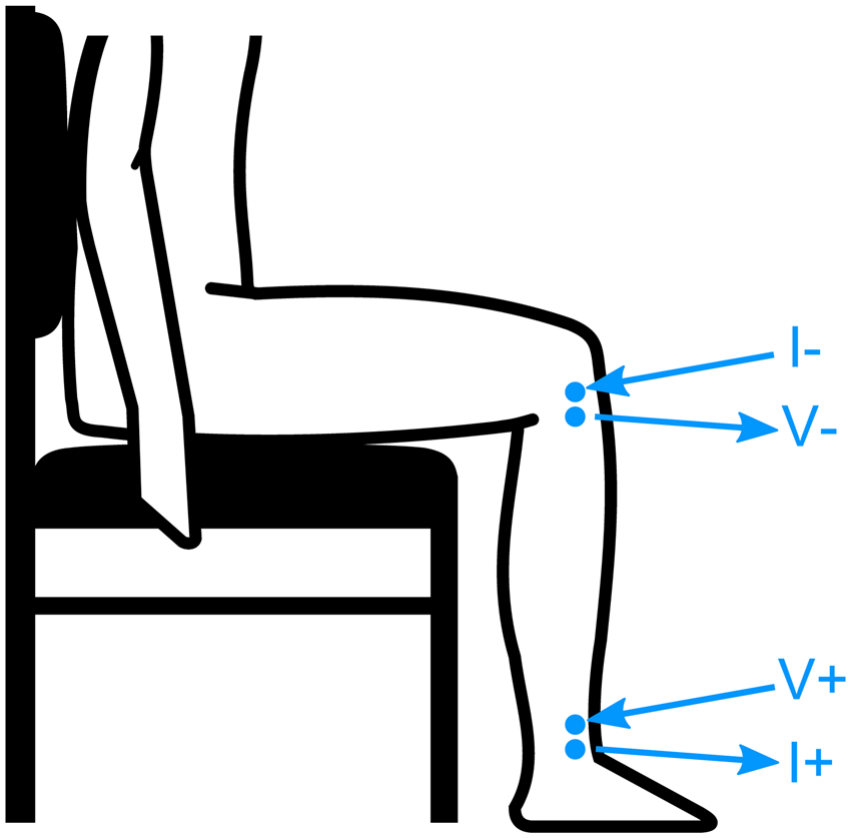
Position of the bioelectrical impedance electrodes on the lower leg. I+ and I− represent electrodes applying current, while V+ and V− are the electrodes measuring the voltage across the lower leg.

Surface electrodes were placed on the skin at the top and bottom of the lower leg (ankle and knee) as illustrated in Fig. 6 using hypoallergenic adhesive tape. At the ankle, voltage measuring (V+) electrode was placed just below the lateral malleolus. At the knee the voltage measuring (V−) electrode was placed between the fibula head and tibial tubercle. Current injecting electrodes labeled I+ and I− were positioned 1-inch outside the voltage measuring electrodes and injected a small amplitude (400 μA), high frequency (25 kHz) current into the leg. Voltage drop across the leg measured by V+ and V− electrodes were used to calculate resistance used in equation 1.

Using a measuring tape, segment length was measured as the distance between the measurement electrodes. Circumferences of the ankle and knee were measured at the level of the measurement electrodes at the start and end of sitting. A line was drawn around the ankle, and knee to ensure measurements were consistent when repeated. Using the same method, calf measurements were also made around the largest part of the calf at the start and end of sitting, for use as an alternative measure of fluid accumulation.

### Electrical Stimulation Protocol

Calf muscle ES was performed using a programmable 4-channel neuromuscular stimulator (Compex Motion, Switzerland). Electrical current was delivered transcutaneously to the gastrocnemius muscle groups of both legs using 5 cm by 9 cm StimTrode neurostimulation electrodes (Axelgaard Manufacturing, California, USA) positioned on the calf over the approximate position of the motor points^44^. The stimulation protocol used an asymmetrical biphasic waveform with 40 Hz frequency, 300 μs pulse duration, at the maximally tolerated stimulation amplitude (typically between 20 and 40 mA). The duty cycle was 2 seconds on, followed by 2 seconds off. The left and right gastrocnemii were stimulated out of sync, such that when the left gastrocnemius was contracted the right gastrocnemius was relaxed and vice versa. A sham electrical stimulation protocol was also utilized as a control. The sham ES protocol was identical to the active ES protocol described in terms of the current waveform used and electrode type and position. However, the main difference was the amplitude of the electrical current which was set much lower in the sham ES protocol, at a level that elicited the sensation of electrical current travelling through the lower leg, without causing calf muscle contraction.

### Modeling Temporal Fluid Data

Temporal fluid data in the active and sham ES conditions consisted of two distinct portions: an initial portion and a latter portion which differed by their apparent slopes. In addition, the initial portions appeared to increase according to an exponential decay function, whereas the latter portions appeared linear. Similarly, the difference in fluid volume between the active and sham ES conditions also consisted of two distinct portions. Each portion was modeled with both a linear and exponential decay function, described in equations 2 and 3; where *LFV* is the leg fluid volume (ml), *m* is the slope of the line (ml/min), *b* is the y-intercept (ml), *t* is time (minutes), τ is the time constant (minutes), and *LFV*_*end*_ is the expected final value of the leg fluid volume (ml). The model with the best fit was selected to describe each portion of data.

2 $$LFV(t)=mt+b$$

3 $$LFV(t)=LFVend(1-{e}^{-\frac{t}{\tau }})$$

### Change in Leg Fluid Volume per Contraction

The change in leg fluid volume per contraction was estimated using the change in resistance (ΔR) per contraction. Illustrated in Fig. 4, this metric was computed by subtracting the mean resistance during each contraction (active) from the resistance when contraction ended (inactive). This was performed for every contraction over the 150 minute seated period, from which mean ΔR per contraction was computed, as described in equation 4, where N is the total number of contractions. Given that contractions occurred every two seconds, each participant experienced approximately 4500 contractions over the seated period.

4 $${\rm{\Delta }}R=\frac{1}{N}\sum _{n=1}^{N}{[\overline{active}-\overline{inactive}]}_{n}$$

### Study Protocol

The study was a randomized, single-blind double crossover protocol. Data collections were completed over two days set one week apart in a quiet, temperature-controlled room following abstention from alcohol and caffeine. Experiments began in the morning between 10 and 11AM for all participants. On the first morning, participants were randomly assigned to sham or active ES. On each morning, surface electrodes were placed on the legs to collect bioelectrical impedance data, as illustrated in Fig. 6. Participants initially lay supine for 30 minutes to ensure all participants began sitting with a similar level of leg fluid. Next, participants sat for a two-and-a-half hour period with either active or sham ES being applied to their calf muscle. Participants sat with their legs still, but were free to move their upper body.

### Data Acquisition and Analysis

Bioelectrical impedance data was collected continuously at 1.25 kHz using Biopac data acquisition system (MP150, California, USA) and imported into Matlab (2014a, Massachusetts, USA) for pre-processing. Signals were manually adjusted for motion artifacts based on documented events of movement or seat adjustment occurring during data collection. Signals were then averaged every minute to reduce variability in the signal due to noise or other artifacts.

Bioelectrical impedance signals were converted to fluid volumes using Equation 1. To account for changing leg circumferences over the seated period, circumferences measured before and after the seated period were used to interpolate all values in between. Difference in leg fluid accumulation between the active and sham ES study conditions were computed by subtracting the fluid data from each participant in the two study conditions. The effect size of the difference in leg fluid was calculated by dividing the mean difference at each time point by the standard deviation at the same time point.

Statistical differences in baseline values of leg fluid volume, and calf circumference between study conditions were analysed using the paired t-test (or Wilcoxon signed-rank test for non-normally distributed data). A repeated measures ANOVA was used to compare changes in leg fluid volume, and calf circumference from start to end of sitting, between study conditions. The ANOVA included two factors: timing (start and end) and study condition (sham ES or active ES). The interaction effect tested differences in the change in leg fluid or calf circumference from the start to the end of sitting between active and sham ES conditions. Lastly, the paired t-test (or Wilcoxon signed-rank test for non-normally distributed data) was used to test if the change in leg fluid or calf circumference from start to end of sitting within each study condition was statistically significant.

Data distributions were assessed using the Shapiro-Wilk test of normality and considered non-normally distributed if the null hypothesis was rejected at a significance level of p < 0.05. Correlations were performed using Pearson correlations with normally distributed data and Spearman’s rank correlation with non-normally distributed data. A p-value < 0.05 was considered statistically significant. All data are reported as the mean ± SEM.
